# A national multi centre pre-hospital ECPR stepped wedge study; design and rationale of the ON-SCENE study

**DOI:** 10.1186/s13049-024-01198-x

**Published:** 2024-04-17

**Authors:** Samir Ali, Xavier Moors, Hans van Schuppen, Lars Mommers, Ellen Weelink, Christiaan L. Meuwese, Merijn Kant, Judith van den Brule, Carlos Elzo Kraemer, Alexander P. J. Vlaar, Sakir Akin, Annemiek Oude Lansink-Hartgring, Erik Scholten, Luuk Otterspoor, Jesse de Metz, Thijs Delnoij, Esther M. M. van Lieshout, Robert-Jan Houmes, Dennis den Hartog, Diederik Gommers, Dinis Dos Reis Miranda

**Affiliations:** 1 Department of Intensive Care, Erasmus University Medical Centre, Dr. Molewaterplein 40, Rotterdam, 3015 GD the Netherlands; 2 Department of Anaesthesiology, Erasmus Medical Centre, Rotterdam, 3015 GD the Netherlands; 3 Ministry of Defence, Royal Netherlands Air Force, Breda, 4820 ZB the Netherlands; 4 Helicopter Emergency Medical Services, Trauma Centre Zuid-West Nederland, Erasmus University Medical Centre, Rotterdam, 3045 AS the Netherlands; 5 Helicopter Emergency Medical Services, Netwerk Acute Zorg Noordwest, Amsterdam University Medical Centre, Amsterdam, 1081 HV the Netherlands; 6 Helicopter Emergency Medical Service, Radboud University Medical Centre, Nijmegen, 6525 GA the Netherlands; 7 Department of Anaesthesiology, Maastricht University Medical Centre, Maastricht, 6229 HX the Netherlands; 8 Helicopter Emergency Medical Service, University Medical Centre Groningen, Groningen, 9713 GZ the Netherlands; 9 Department of Intensive Care, Amphia Hospital, Breda, 4818 CK the Netherlands; 10 Department of Intensive Care Medicine, Radboud University Medical Centre, Nijmegen, 6525 GA the Netherlands; 11 Department of Intensive Care Medicine, Leiden University Medical Centre, Leiden, 2333 ZA the Netherlands; 12 Department of Intensive Care Medicine, Amsterdam University Medical Centre, Amsterdam, 1105 AZ the Netherlands; 13 Department of Intensive Care, Haga Teaching Hospital, the Hague, 2545 AA the Netherlands; 14 Department of Critical Care, University Medical Centre Groningen, Groningen, 9713 GZ the Netherlands; 15 Department of Intensive Care, St. Antonius Hospital, Nieuwegein, 3435 CM the Netherlands; 16 Department of Intensive Care, Catharina Hospital, Eindhoven, 5623 EJ the Netherlands; 17 Department of Intensive Care, OLVG, 1091 AC Amsterdam, the Netherlands; 18 Department of Intensive Care, Maastricht University Medical Centre, Maastricht, 6229 HX the Netherlands; 19 Trauma Research Unit, Department of Surgery, Erasmus MC, University Medical Centre Rotterdam, Rotterdam, 3015 GD the Netherlands

**Keywords:** Out-of-hospital cardiac arrest, Extracorporeal membrane oxygenation, Cardiopulmonary resuscitation, Advanced cardiac life support

## Abstract

**Background:**

The likelihood of return of spontaneous circulation with conventional advanced life support is known to have an exponential decline and therefore neurological outcome after 20 min in patients with a cardiac arrest is poor. Initiation of venoarterial ExtraCorporeal Membrane Oxygenation (ECMO) during resuscitation might improve outcomes if used in time and in a selected patient category. However, previous studies have failed to significantly reduce the time from cardiac arrest to ECMO flow to less than 60 min. We hypothesize that the initiation of Extracorporeal Cardiopulmonary Resuscitation (ECPR) by a Helicopter Emergency Medical Services System (HEMS) will reduce the low flow time and improve outcomes in refractory Out of Hospital Cardiac Arrest (OHCA) patients.

**Methods:**

The ON-SCENE study will use a non-randomised stepped wedge design to implement ECPR in patients with witnessed OHCA between the ages of 18–50 years old, with an initial presentation of shockable rhythm or pulseless electrical activity with a high suspicion of pulmonary embolism, lasting more than 20, but less than 45 min. Patients will be treated by the ambulance crew and HEMS with prehospital ECPR capabilities and will be compared with treatment by ambulance crew and HEMS without prehospital ECPR capabilities. The primary outcome measure will be survival at hospital discharge. The secondary outcome measure will be good neurological outcome defined as a cerebral performance categories scale score of 1 or 2 at 6 and 12 months.

**Discussion:**

The ON-SCENE study focuses on initiating ECPR at the scene of OHCA using HEMS. The current in-hospital ECPR for OHCA obstacles encompassing low survival rates in refractory arrests, extended low-flow durations during transportation, and the critical time sensitivity of initiating ECPR, which could potentially be addressed through the implementation of the HEMS system. When successful, implementing on-scene ECPR could significantly enhance survival rates and minimize neurological impairment.

**Trial registration:**

Clinicaltyrials.gov under NCT04620070, registration date 3 November 2020.

## Background

Of the patients who survive an Out-of-Hospital-Cardiac-Arrest (OHCA), approximately 90% achieves return of spontaneous circulation (ROSC) within 15 min [1]. In case of refractory arrest, often defined as ROSC not obtained within 20 min, favourable neurological outcome is uncommon (OR 0.03) [1–3]. Currently, patients failing to reach ROSC are either declared deceased on-site or transported to the hospital for further treatment, including potential Extracorporeal Cardiopulmonary Resuscitation (ECPR) eligibility. The application of ECPR is a rapidly growing, and although ECPR could be an effective treatment, its success relies heavily on being initiated in time [4].

**Fig. 1:**
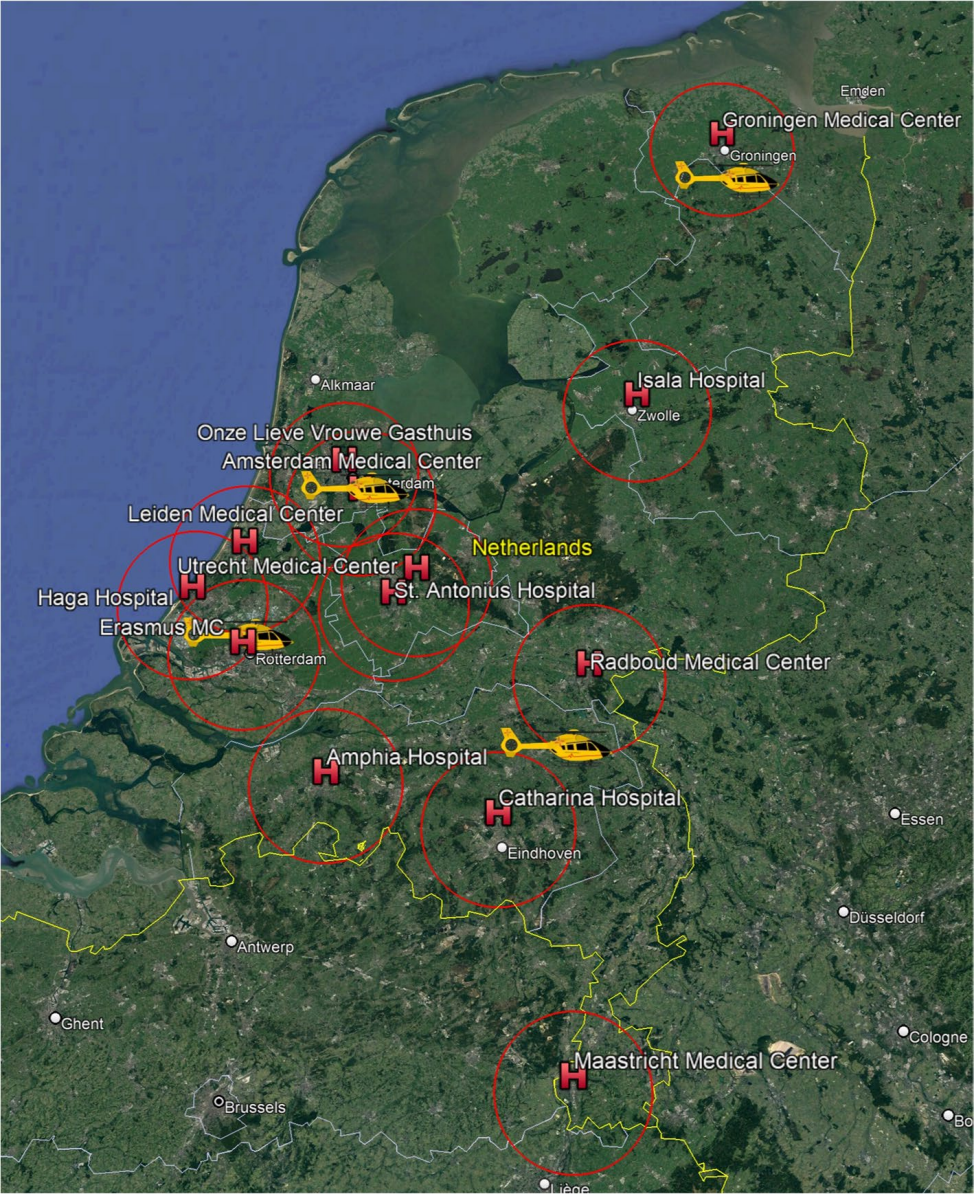
The 10 ECPR centres with their 30 min radius

Multiple studies have demonstrated that in patients receiving ECPR for refractory OHCA, a cardiopulmonary resuscitation duration of less than 40 min was strongly associated with better survival with minimal neurological impairment [5–8]. Suboptimal thoracic compression during transportation further reduces survival probability, leading to prolonged low-flow times [9], especially if the cardiac arrest occurs far from an ECPR centre (Fig. 1).

Initiating ECPR at the cardiac arrest scene could potentially overcome both problems by reducing the duration of the low-flow state and limiting thoracic compression during transport. Performing ECPR at the scene will enlarge the potential patient pool to include individuals who might not meet the criteria for in-hospital rescue therapy due to the prolonged period of low flow caused by transportation delay. With the use of the Helicopter Emergency Medical Services (HEMS), the geographic range can be extended while maintaining a short low-flow time. This perspective is the cornerstone of the ON-SCENE study.

In the Netherlands, HEMS teams consist of an anaesthesiologist or trauma surgeon, an emergency or ambulance registered nurse, and a pilot. Their primary goal is to provide specialized medical care to trauma and non-trauma patients throughout the country with an arrival at scene after approximately 11 min. Dispatching the HEMS simultaneously with the first responders after OHCA, could potentially shorten time between collaps and initiation of ECMO bloodflow which in turn could significantly improve the chance of survival compared to either conventional resuscitation without ECPR and ECPR initiated in-hospital [10].

We therefore designed and set up the ON-SCENE study to investigate the effect of equipping the HEMS with prehospital ECPR facility on hospital survival. In this HEMS setting, a nationwide coverage for prehospital ECPR in a timely manner would be achieved. The hypothesis of this study is that implementing nationwide on scene ECPR in patients with OHCA results in shorter low-flow time in comparison to regular care, which leads to improved survival rates and less neurological impairment. Despite the increased costs for delivering ECPR on scene, it is likely to be cost-effective.

## Methods

### Trial design

This is a nationwide multicentre, prospective, interventional, non-randomised stepped wedge study that evaluates the effect of prehospital ECPR in patients with witnessed OHCA between the age of 18 and 50 years old versus conventional HEMS care without ECPR. The study started end of 2021 and is scheduled to finish by the end of 2025.

### Eligibility criteria

Patients with a witnessed OHCA between the age of 18 and 50 years old and an initial presentation of ventricular fibrillation/pulseless ventricular tachycardia, as well as pulseless electrical activity with a high suspicion of pulmonary embolism are eligible for inclusion. Detailed inclusion and exclusion criteria are listed in Table 1. The eligibility criteria aim to identify patients with a cardiac arrest that have treatable underlying causes.

**Table 1:** Inclusion and exclusion criteria

**Inclusion criteria**	**Exclusion criteria**
Witnessed out of hospital cardiac arrest	ROSC ≤ 20 min
≥ 18 ≤ 50 years (known or estimated)	Expected time from collapse to arrival at an ECPR centre with a direct available ECPR team is less than 30 min.
Initial rhythm of (p)VT /VF or suspected PE	EtCO_2_ et < 1.2 kPa (10 mmHg) during CPR
Refractory cardiac arrest ≥ 20 min ≤ 45 min	No clear ultrasound visualisation of either the femoral artery or the femoral vein.
	**Following initial inclusion, the following patients will be eliminated as soon as the following details are made available**
	Active malignancy
	Cerebral vascular accident < 6 weeks
	Patients with a “do not resuscitate” order, which was not known at time of the arrest

*(p)VT* (pulseless) Ventricular Tachycardia, *VF* Ventricular Fibrillation, *PE* Pulmonary Embolism, *ROSC* Return of Spontaneous Circulation, *ECPR* Extracorporeal Cardiopulmonary Resuscitation, *CPR* Cardiopulmonary Resuscitation, *kPa* Kilopascal

Witnessed arrest is defined as patients who have been last seen well less than 5 min before or show signs of life, meaning extremity movements, normal pupillary reflexes, or gasping during CPR. Whenever the exact age is unknown at the time of care, it is estimated by HEMS. If the patient later on found to be outside the inclusion range of age after cannulation, they will not be excluded.

The time of start of arrest to expected time of arrival at the hospital should be longer that 30 min to be eligible to prehospital ECPR. Furthermore, it should be assumed that the ECPR team would be ready on the ER when the patient arrives. If this is not expected, patient will then receive prehospital ECPR. Only very few patients are so close to the hospital that start of arrest to expected time of arrival hospital is less than 30 min.

### Allocation of patient

**Fig. 2:**
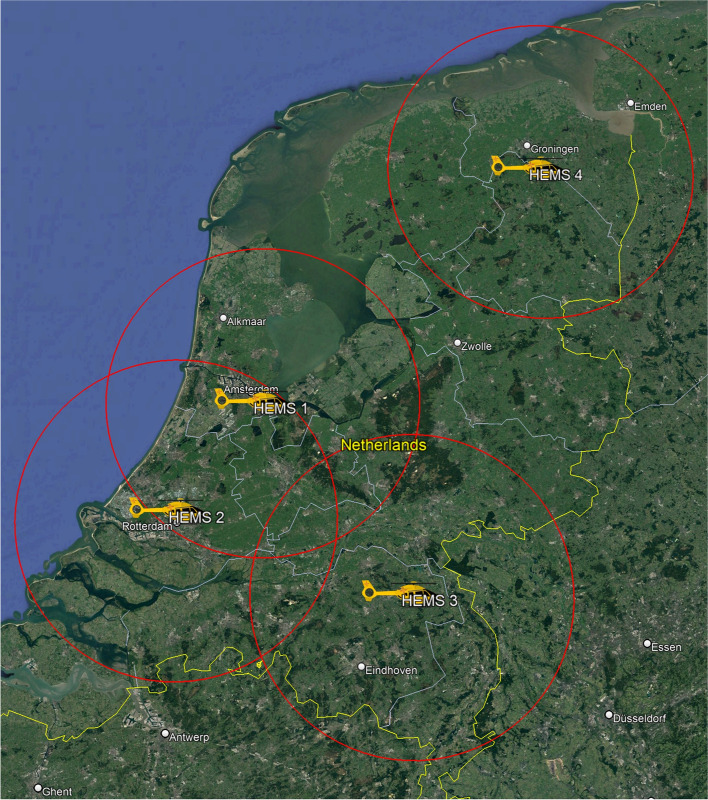
HEMS Region with their 20 min geographic radius. HEMS: Helicopter Emergency Medical Services

There are four HEMS stations serving the Netherlands, situated in Groningen, Rotterdam, Amsterdam, and Nijmegen (Fig. 2) which cover the majority of the country. Before this study, HEMS crews are not routinely involved in adult OHCA cases. In this study, HEMS will be dispatched simultaneously with the emergency medical services to every witnessed OHCA involving patients suspected to be between the ages of 18 and 50 years. As ECPR will not be offered in a regional manner (as it is typical nowadays) but on a national level, we were cautious not to overwhelm the system. To limit the number of ECPR cases, we exclude patients older than 50 years. A computer-aided system will assist the dispatcher in the central dispatch centre to determining patient eligibility. In the intervention group, the HEMS will be equipped with ECPR equipment. In the control group, HEMS will also be deployed, but without ECPR equipment. This is done to prevent a second treatment effect in the ECPR group, being the assistance of the HEMS team on scene.

After start of the study some large dispatch centres still seemed not ready to dispatch the HEMS because of legal uncertainties. Therefore, just after start of the study, an amendation to extend the study period was filed. To prevent protocol violation, dispatch centres who weren’t ready at the start of the study were excluded until being completely ready to comply with the study protocol.

### Training of HEMS personnel

**Fig. 3:**
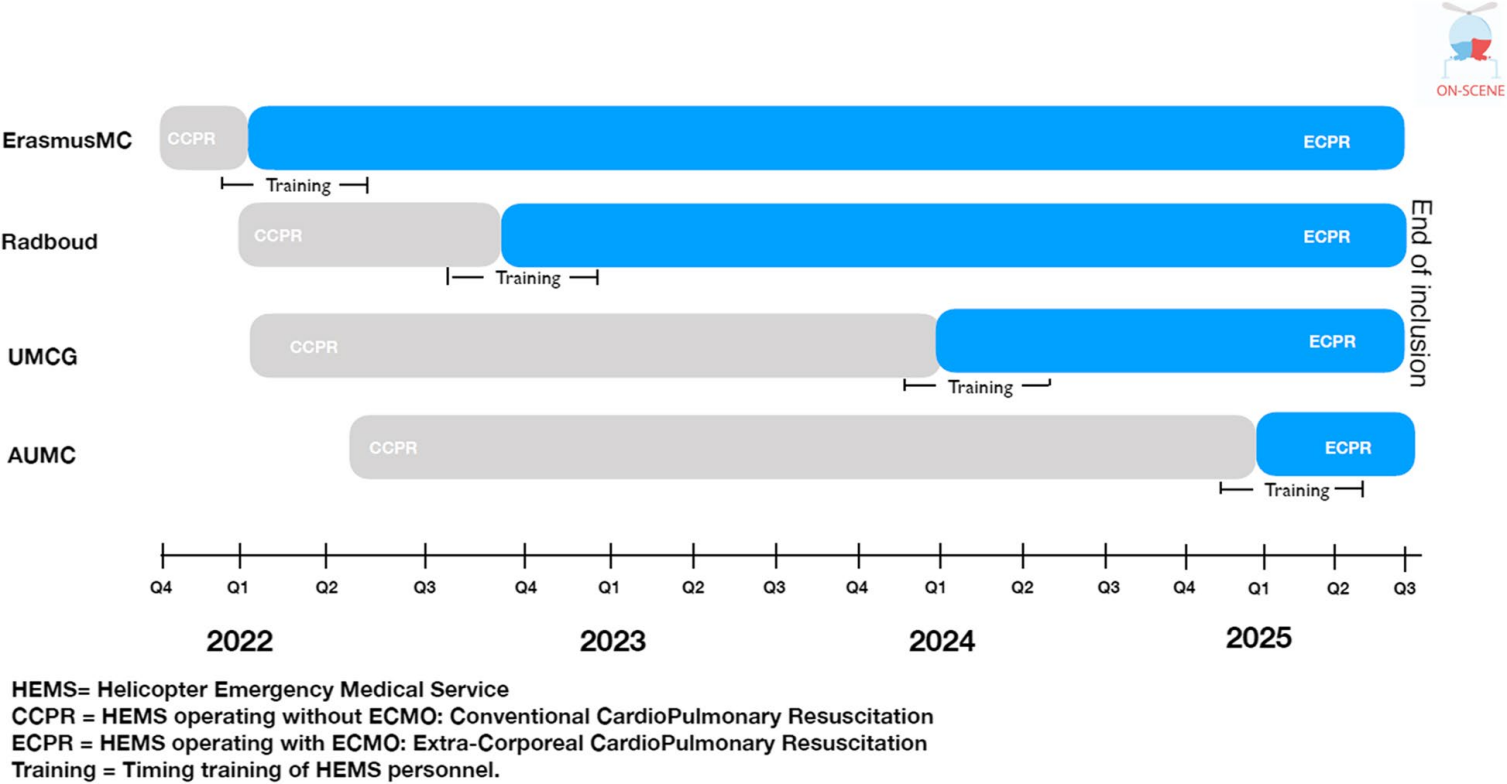
Schematic timetable of the stepped-wedge study design. ECPR experienced personnel and non-ECPR experienced personnel). HEMS: Helicopter Emergency Medical Service. CCPR: HEMS operating without ECMO: Conventional CardioPuImonary Resuscitation. ECPR: HEMS operating with ECMO: Extra-Corporeal CardioPulmonary Resuscitation Training: Timing training of HEMS personnel

Training is key for all high-stakes procedures, especially under time constraints. Given that the capacity to train all HEMS teams at the same time is limited, a stepped-wedge design was chosen (Fig. 3) [11, 12].

This design also prevents cross-over to the intervention arm in young patients who fail to achieve return of spontaneous circulation, thereby improving the study’s quality.

To train the HEMS personnel, a mobile high-fidelity ECMO training simulation centre that offers team-based training in ECPR cannulation and management will be used. This training program is used for years to train thoracic intensivist for achieving a 24/7 in-hospital ECPR service.

Each HEMS crew member will undergo a comprehensive training program led by a minimum of three trainers for every six members, consisting of theoretical and practical elements. The training will include a-4 h E-learning sessions followed by a 6-h plenary sessions. The practical component of the training will include a 2 day training period involving hands-on experience, with each crew member performing at least 10 cannulations on a specially designed mannequin. These cannulations will be executed in high-fidelity simulations, gradually increasing in complexity and incorporating challenging scenarios that simulate uncomfortable positions and environments mirroring real-life situations. The initial training is signed off by a mannequin cannulation in a challenging condition, without errors and in a timely manner. After completion, HEMS stations will be equipped with ECPR facilities. Continuous training will follow for 6 months on a weekly basis, then ongoing sessions every 3 months. Beyond cannulation, training will encompass ECMO management and troubleshooting, facilitated by experienced perfusionists and ECMO intensivists, using real in-hospital patient to enhance skills and maintain proficiency.

To provide ongoing support to HEMS crew during and after cannulation, a 24-h telemedicine system is established. Immediately after cannulation and before transport, a mandatory live videocall is made with ECMO physicians and perfusionists.

### Treatment in the intervention group, prehospital ECPR

In the intervention group, HEMS personnel will initiate ECMO cannulation after 20 min of refractory cardiac arrest. The decision to proceed with the cannulation is made solely by the HEMS physician on the basis of the available information regarding the inclusion and exclusion criteria. Since the time it takes for ECMO initiation significantly impacts outcomes, HEMS will collect both the arrival time at the patient location and the time of ECMO flow.

Vascular access will be ascertained through an ultrasound guided puncture of the femoral artery and vein. Insertions of an 25 French venous cannula and a 17 French arterial cannula is performed followed through a Seldinger procedure using an Amplatz Super Stiff™ (with J-tip) guidewire. If they estimated weight is below 50 kg, an 21 French venous and 15 French arterial cannula is inserted(Getinge, Germany). The ECMO machine will then be connected to the cannulas to restore blood flow mechanically, after which chest compressions can be stopped. Target ECMO flow will be between 3 and 4 L per minute and a mean arterial pressure (MAP) of at least 75 mmHg measured with an invasive femoral arterial line placed in the other groin. Arterial blood pressure will be measured using a disposable Compass pressure device (Centurion Medical Products, USA). The ECMO oxygen fraction will initially be set at 0.6 with a standard sweepflow of 2L/min. Ventilation will be maintained at a tidal volume of 4 ml /kg of ideal bodyweight and a respiratory rate of 6 per minute to prevent a rapid decrease in PaCO2. Saturation levels will be measured on the right hand to detect a possible harlequin syndrome. The cannulas will be fixated, and the patient will be transported by ambulance to the nearest hospital providing ECMO care. Standard post-resuscitation care will be provided in the hospital.

### Treatment in control group, conventional CPR

The control group will receive standard advanced life support. In contrast to the current situation, the HEMS team will also be dispatched in addition to the standard dispatch of two ambulance crews. If possible, eligible patients in the control group will still be transported to a hospital with ECPR capabilities.

### Post resuscitation care

Upon admission, all patients will be transferred to the hospital’s ICU department and treated per accepted guidelines. During the first 24 h, the targeted temperature range will be maintained between 33 and 36 degrees Celsius. In addition, patients will undergo coronary angiography as soon as possible according to national guidelines if indicated.

The nationwide approach will ensure uniformity in care cessation protocols across all participating hospitals. Prognostic evaluation of post-anoxic coma and termination of care will be performed according to the national guideline, which are based on the European Resuscitation Council (ERC) and European Society of Intensive Care Medicine Guidelines [13, 14]. After 24 h, comatose patients without significant sedation can be withdrawn from life sustaining therapy if one of the criteria as described in Table 2 is met. Both the ECPR group and the control group will be evaluated on identical criteria.

**Table 2:** Criteria to withdraw from life sustaining therapy after 24 h in comatose patients

Dilated, non-reactive pupils after 24 h *OR*
Bilateral absent N20 response on the somato-sensory evoked potentials* OR*
EEG showing highly malignant patterns after 24 h (low voltage (< 20uV)* OR*
Identical bursts suppression pattern* OR*
Generalized periodic discharges on an iso-electric ground pattern* OR*

### Devices used

At each HEMS station, a helicopter and ground vehicle are both equipped with a CardioHelp device (Getinge, Germany) for ECPR. If the weather conditions do not permit flying or the incident is close to the HEMS station, dispatch is performed by car. The same device will be used in post-resuscitation care.

### Outcome

#### Primary outcome measure

The primary outcome measure is survival at hospital discharge.

#### Secondary outcome measure

Secondary outcome measures are good favourable neurological outcome, quality-adjusted life years and health care costs.

Good neurological outcome is defined as a CPC (Cerebral Performance Categories Scale) score of 1 or 2 at 6 and 12 months. Quality-adjusted life year (QALY) is calculated at 6 and 12 months after discharge using the EQ-5D-5L tool [15]. Costs are calculated based on the use of disposables, depreciation of the ECMO machines, flight time of the helicopter and costs of institutionalisation. Healthcare costs will be calculated using the iMTA Medical Consumption Questionnaire (iMCQ) [16] and the iMTA Productivity Cost Questionnaire (iPCQ) [17]. Costs are calculated in the first 6 and 12 months after cardiac arrest.

#### Other data collected

In addition to the outcome measures, the following data will be collected:

- Baseline variables like gender, age, comorbidity.
- ALS-related variables: first observed rhythm, end-tidal CO_2_ before cannulation, motor score before cannulation, signs of life, total mg of adrenaline use, use of mechanical CPR.
- ECPR-related variables: duration of low-flow state, MAP before transport to hospital, ECMO blood flow before transport to hospital, any complication due to ECPR.
- In hospital-related variables: Systolic and diastolic blood pressure at and after 12 h arrival of emergency department, lactate at and after 12 h arrival of ED, use and type of unloading device, number of days at ECMO and ventilator.

### Ethical considerations

The study is approved by the Medical Research Ethics Committee of the Erasmus MC and other participating ECMO centres. This study is registered at clinicaltrials.gov under NCT04620070, registration date 3 November 2020.

Patients will be screened for inclusion after arrival of the HEMS team. The patients eligible for this study are unconscious. The study intervention is an emergency intervention that must be applied (or not) without delay and fulfils the ethical requirement of clinical equipoise. Eligible patients have a high risk of dying, and their legal representatives may be in a disturbed mental state, making an immediate informed decision impossible. If the patient’s consciousness recovers, or if the legal representative remains unable to communicate, the investigator or supervising doctor will inform the patient or seek deferred proxy consent for the use of study data.

### Statistical consideration

#### Sample size

In the intervention group, the assumption is that ECPR, being initiated within 35 min after onset of cardiac arrest, thereby would result in a hospital survival of 35%. Due to an anticipated learning curve for the HEMS crews, a longer time to cannulation can be expected. The estimation is that this will cause a decrease of survival to 30% in the first 3 months.

In the control group, we assume that 30% of the patients with refractory arrest are transported to the hospital to receive ECPR and that 70% is not transported to an ECPR facility during cardiac arrest. Assumed hospital survival with ECPR in the hospital is 26% (data from Erasmus MC, Rotterdam the Netherlands) and 16% if the patient is not transported to an ECPR facility.

With the different scenarios described above, sample size was calculated using a simulation approach (R package contrast). Anticipating a possible loss of power caused by the stepped wedge design, 175 patients will be included in each study arm to detect the survival difference in mentioned above with 90% power and alpha 0.05. In total 390 patients will be included.

By extrapolating from the Amsterdam ‘ARREST’ database, we estimate that 450 patients in a year of below 50 years will have a witnessed OHCA. Assuming that 25% will fulfil the inclusion criteria, we expect that a maximum of 100 patients a year are eligible for the study. Therefore we assume that a period of 3.5 years should be sufficient to reach the needed number of patients for our study.

### Statistical analysis

#### Primary and secondary

The primary outcome measure, hospital survival, will be analysed by descriptive statistics per group and percentage of total. Differences in hospital survival between groups (intervention and control group) will be tested using univariate analysis (intention to treat) by means of a Chi-square test.

As a result of the cluster effect, the primary analysis using unadjusted Chi-square test might be biased. To asses for this and other cofounders a secondary, multivariable analysis will be performed using Generalized Estimating Equations (GEE). Survival will be a dependent variable, prehospital ECPR will be included as covariate. No prehospital ECPR will be the reference category. Baseline and EMS/dispatch related variables that may potentially confound the association between prehospital ECPR and outcome will be included in this model as covariate. Known potential confounders include age, gender, time to ambulance arrival on scene, low-flow time, known medical cardiovascular conditions having an increased risk of sudden cardiac arrest. If these variables produce a *p*-value < 0.2 in a univariate comparison between the two groups, they will be included in the regression model.

Secondary outcome like survival with good neurological outcome (defined as CPC 1–2) at 6 and 12 months will be analysed using a logistic mixed regression analysis for between the two groups.

#### Subgroup

A subgroup analysis will be performed dividing the control groups: one group who received no ECPR and one group who received ECPR after transportation to the hospital. Both therapies in the control group will be compared (using GEE) with the intervention group: prehospital ECPR. Modelling strategy will be the same as described for the primary analysis.

Other a priori defined analytic subgroups are initial cardiac rhythm (shockable vs non-shockable), time to start ECMO flow (< 40 min vs ≥ 40 min) and presence of signs of life during CPR (yes vs no).

Also, historical comparison will be made. Before the ON-SCENE study, dispatch centres were not used to activate the HEMS in an adult cardiac arrest. From the Dutch ARREST database, sequential OHCA patients will be selected who fulfil the ON-SCENE Study inclusion criteria in the timeslot before start of the ON-SCENE study. Patients in which HEMS were activated will be excluded. Hospital mortality will be the primary outcome and secondary the CPC score after 6 and 12 months.

## Discussion

This manuscript describes the design of the ON-SCENE study, being a nationwide HEMS based initiation of ECPR on the site of cardiac arrest. This study will address the question whether HEMS equipped with prehospital ECPR facility will improve outcomes for cardiac arrest patients.

OHCA continues to impose a significant social and economic health burden on society [18]. While survival of OHCA patients increased tremendously from 13.7% in 2000 to 22.3% in 2009 [19], with bystander CPR and AED use being the most important improving factors [20, 21], survival rates have plateaued since then [22].

Multiple studies have shown a strong relation between low-flow time and survival [3, 23, 24]. ECPR is proposed as a way to further improve survival rates by means of shortening the low-flow time in patients with refractory cardiac arrest. A meta-analysis analyses and multiple observation studies indicate that ECPR does indeed significantly improve outcomes [23, 25, 26] and may be considered according to the ERC Guidelines [27]. However ECPR can only have a meaningful impact if the low-flow time remains short.

The first randomized ECPR trial was the ARREST trial, which randomised OHCA patients to ECPR or standard care and found that the ECPR group had a 43% discharge survival rate compared to 7% in the standard group [28]. However, the trial was prematurely stopped after only 30 patients were included because the predefined criteria of superiority were met. The small sample size makes the external validation of the study difficult to interpret.

The second trial, the Prague OHCA study, a single-centre trial with 256 adult patients facing refractory OHCA presumed to be cardiac in origin, explored two strategies: an invasive approach (*N* = 124) and a standard strategy (*N* = 132) maintaining on-scene ACLS [29]. The primary outcome was survival with a good neurologic outcome at 180 days. While the primary outcome of 180-day survival with good neurological function showed no significant difference between the groups (31.5% vs 22.0%; *P* = 0.09), a secondary analysis revealed the effectiveness of ECPR for resuscitated patients at hospital arrival [30]. Factors contributing to less favorable outcomes included the presence of non-shockable rhythms in 39.1% of patients, known been a poorer prognosis compared to shockable rhythms [31–34]. This observation was also reinforced by a post-hoc analysis within the same study group [35]. Consequently, the emphasis on patients with initial shockable rhythms becomes argumentative.

The latest multicentre RCT trial, INCEPTION, randomized witnessed OHCA patients with shockable rhythms to either standard ACLS or ECPR upon arrival at ten ECPR centres in the Netherlands [36]. Results showed no significant difference in survival with a favourable neurological outcome (CPC score of 1 or 2) among the 160 patients (20.0% vs 16.0%; *P* = 0.518). The time from cardiac arrest to ECMO flow, averaging 74 min, was notably longer than the 59-min average in the ARREST trial, which demonstrated a positive effect of ECPR. The ON-SCENE study aims to reduce this low flow time and enhancing outcomes for these patients by introducing ECPR at the cardiac arrest site.

The Netherlands is an excellent region to test the efficacy of pre-hospital ECPR, it has a homogenous and well-organised pre-hospital and post-cardiac arrest care system. AEDs and bystander CPR systems are widely available and are activated by a layman system. This resulted in a 100% coverage of initiating of CPR within 6 min after activation [37]. These factors make it possible to implement the concept of ECPR within this developed health system and evaluate its potential impact on post-cardiac arrest care. It is the perfect opportunity to investigate the added value of ECPR.

### Current status of trial

https://onscenetrial.com/

## Data Availability

Not applicable.
